# Methods for the Real-World Evaluation of Fall Detection Technology: A Scoping Review

**DOI:** 10.3390/s18072060

**Published:** 2018-06-27

**Authors:** Robert W. Broadley, Jochen Klenk, Sibylle B. Thies, Laurence P. J. Kenney, Malcolm H. Granat

**Affiliations:** 1 School of Health Sciences, University of Salford, Salford, M6 6PU, UK; s.thies@salford.ac.uk (S.B.T.); l.p.j.kenney@salford.ac.uk (L.P.J.K.); m.h.granat@salford.ac.uk (M.H.G.); 2 Department of Clinical Gerontology, Robert-Bosch-Hospital, 70376 Stuttgart, Germany; jochen.klenk@rbk.de; 3 Institute of Epidemiology and Medical Biometry, Ulm University, 89081 Ulm, Germany

**Keywords:** accidental falls, fall detection, real-world, signal analysis, performance measures, wearable sensors, non-wearable sensors, accelerometers, cameras

## Abstract

Falls in older adults present a major growing healthcare challenge and reliable detection of falls is crucial to minimise their consequences. The majority of development and testing has used laboratory simulations. As simulations do not cover the wide range of real-world scenarios performance is poor when retested using real-world data. There has been a move from the use of simulated falls towards the use of real-world data. This review aims to assess the current methods for real-world evaluation of fall detection systems, identify their limitations and propose improved robust methods of evaluation. Twenty-two articles met the inclusion criteria and were assessed with regard to the composition of the datasets, data processing methods and the measures of performance. Real-world tests of fall detection technology are inherently challenging and it is clear the field is in its infancy. Most studies used small datasets and studies differed on how to quantify the ability to avoid false alarms and how to identify non-falls, a concept which is virtually impossible to define and standardise. To increase robustness and make results comparable, larger standardised datasets are needed containing data from a range of participant groups. Measures that depend on the definition and identification of non-falls should be avoided. Sensitivity, precision and F-measure emerged as the most suitable robust measures for evaluating the real-world performance of fall detection systems.

## 1. Introduction

Falls in older adults and their related consequences pose a major healthcare challenge that is set to grow over the coming decades [1]. Approximately 30 percent of those over the age of 65 experience one or more falls each year, which rises to around 45 percent in those over 80 [2]. Roughly six percent of older adult falls result in fractured bones [3,4]. Falls are estimated to cost the UK over one billion pounds each year, with fractures being the most costly fall related injury [5].

Even when the injuries are not so serious, fallers often struggle to get up unaided [6,7], sometimes leading to a ‘long-lie’ where the faller remains trapped on the floor for an extended period of time. Long-lies can lead to dehydration, pressure sores, pneumonia, hypothermia and death [8,9,10,11]. Further to the physical consequences, the fear of falling can impact on older adults’ quality of life. A fear of falling is associated with a decline in physical and mental health, and an increased risk of falling [12]. Estimates suggest that between 25 and 50 percent of older adults are fearful of falling and half of these will limit their activities as a result [13,14].

One method used to address the severe consequences associated with falling is the use of a push button alarm system, which can ensure help is received quickly, and reduce the risk of a long-lie. However, studies have shown that 80 percent of fallers do not or cannot activate their alarm following a fall, meaning an alternative approach is needed [6,15]. As a result, there has been extensive research into automatic detection of falls and a broad range of approaches have been developed.

In order to understand the efficacy of the automated fall detection systems, it is important to have a robust method of testing performance. Key to the assessment of these systems is the evaluation of reproducibility and experimental validity [16]. There are two types of experimental validity: internal and external. Internal validity is the extent to which the results truly reflect the capability of the tested system, and were not influenced by other confounding factors or systematic errors. External validity is the extent to which the results can be generalised across people and environments.

External validity has been a central issue in tests of fall detection systems. The poor external validity has been caused by the use of laboratory simulated falls conducted by young healthy adults. The accidental, unexpected and uncontrolled nature of a fall makes it challenging to simulate. When a person simulates a fall the movement is expected, deliberate and carried out in a safe space where injury is highly unlikely. Therefore, reflexes to prevent or lessen the severity of the fall are likely to be suppressed leading to a different pattern of movement. When 13 previously published approaches were tested using real-world fall data, the performance was found to be considerably worse (mean sensitivity and specificity of 0.57 and 0.83, respectively) than had originally been reported from testing using simulations (mean sensitivity and specificity of 0.91 and 0.99, respectively) [17].

Despite the challenge associated with simulating falls, the vast majority of studies have used simulated fall data (for recent reviews see [18,19]). The use of laboratory simulated falls has been an accepted approach due to the challenge associated with recording real-world falls. The rarity of falls means that recording them is both costly and time consuming. Bagala et al. [17] estimated that to collect 100 falls, 100,000 days of activity would need to be recorded, assuming a fall incidence of one fall per person every three years. Despite this challenge, the focus is now moving to real-world fall data due to the external validity issues inherent in simulated fall based testing. Real-world data, by its very nature provides high ecological validity and therefore contributes to higher external validity.

The use of real-world data, while a significant step forward, does not make the test robust. Other factors such as cohort selection and size are important for external validity. In addition, the use of real-world data does not increase the internal validity, in fact, the level of variation and abundance of confounding factors creates a greater risk of systematic errors. Therefore, careful consideration and planning of both the data collection and test procedure is vital to ensure the validity of results.

All methods of testing fall detection systems share the same basic framework which shapes the whole method from data collection through to data processing. Therefore, a basic understanding of this framework is needed to understand the best method to evaluate fall detector performance. Fall detection is a case of binary classification; each movement is classified as either a fall (positive case) or non-fall (negative case). For each movement there are four possible outcomes:

- True Positive (TP)—Correctly detected fall
- True Negative (TN)—Non-fall movement not detected as a fall
- False Positive (FP)—Classified as a fall when none occurred
- False Negative (FN)—A fall which was not detected

**Figure 1:**
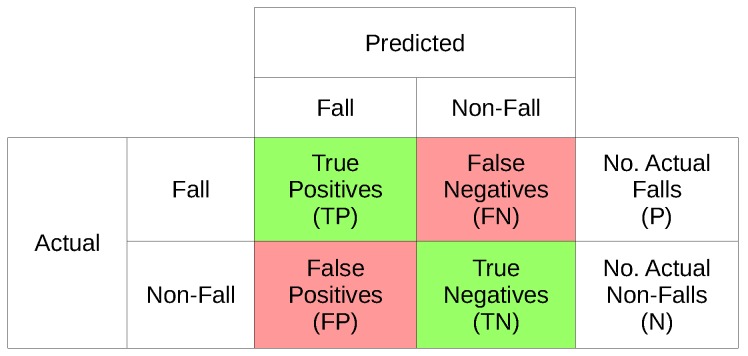
Example confusion matrix.

These four values can be represented as a table comparing the actual data with the system’s predictions, this is known as a confusion matrix (Figure 1). All further measures can be calculated from either a complete confusion matrix or a subset of one. Therefore, studies should aim to collect data and process it in such a way that as many of these four values as possible can be calculated.

The aim of this review is to identify the methods which have previously been used to evaluate fall detector performance using real-world data and investigate how the differences in these methods of evaluation effect the results. The review covers the methods of data collection and processing as well as the performance measures which have been used for evaluation. In this review, we aim to identify the strengths and limitations of current approaches and propose a more robust approach of evaluation based on the findings.

## 2. Methods

A systematic search was conducted in August 2017 and repeated in March 2018, using the following on-line literature databases: Medline, Cinahl, Pubmed, Web of Science and IEEE Xplore. The search aimed to find all records where a fall detection technology (hardware or software) had been tested using real-world falls. The search strategy used is shown in Table 1. Papers were excluded where no fall detection technology was tested, where tests used fall simulations, or the technology was not aimed at older adults. Only articles available in English were included.

The studies which met the inclusion criteria were assessed with regard to the method used to test the fall detection system. The focus was to assess the robustness of these tests and we therefore did not assess the systems’ design or performance. For a comparison of wearable systems see [17] and for a comparison of non-wearable systems see [20]. All included studies tested fall detection technology using real-world fall data. Where studies reported on both tests using simulated data and tests using real-world data, only the methods used for the real-world portion of the data were considered.

First we reviewed the information studies provided about their participants, how they collected data and the volume of data collected. Next, we examined the methods used to identify fall events and to process the data. Finally, we evaluated the use of each applicable performance measure.

## 3. Results

**Figure 2:**
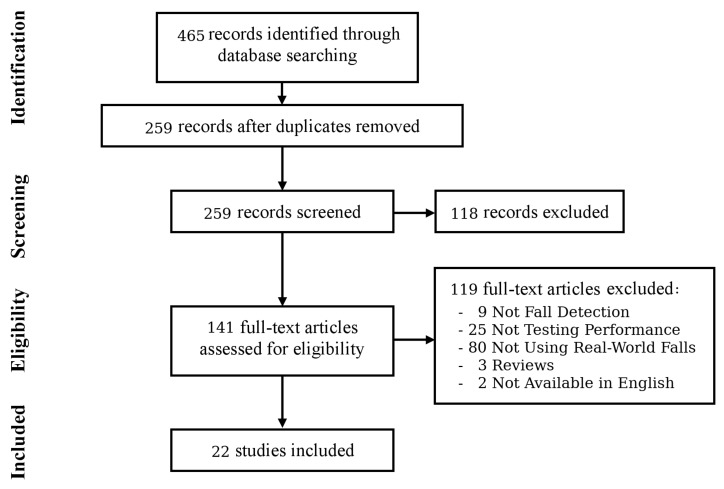
Flow diagram of the systematic search.

The systematic search returned 259 unique records. Following application of the selection criteria, 22 papers were identified for analysis. The full breakdown of the literature identification process, including the reasons for exclusion, is shown in Figure 2. Table 2 provides a breakdown of the 22 included papers with regard to participant groups, devices used, participant numbers, numbers of recorded falls, the quantity and processing of non-fall data and finally, the performance measures reported. The following sections provide further detail to complement Table 2.

### 3.1. Participant Descriptions

The level of detail provided about participants varied considerably. All but three [31,38,40] of the articles stated whether participants were community dwelling, in long-term care or hospital patients. Five articles did not provide any additional descriptive information on the participants [23,24,35,37,40]. The other eighteen articles describe participant’s age, twelve also provide gender information and six provide details of height and weight or BMI [17,25,29,31,32,34]. Four articles provided information on specific medical conditions, three recruited participants with Progressive Supranuclear Palsy [17,21,36] and one included a single older adult with Parkinson’s disease [31]. Lipsitz et al. [34] provided the most in-depth description with a breakdown of the proportion of participants with a range of 21 comorbidities. Eight articles reported results of mobility assessments [21,27,28,29,30,31,33,38], three articles provided information on walking aid use [20,27,28] and three articles additionally reported results of cognitive assessments [29,30,33]. None of the other 15 articles reported standardised measures of cognitive or mobility status.

### 3.2. Method of Data Collection

All studies used the same general approach of monitoring participants with one or more sensor devices. Studies can be classified into two main categories, those using wearable technology (e.g. accelerometers or gyroscopes) and those using non-wearable technology (e.g. fixed cameras or Kinect sensors). Both approaches have advantages and disadvantages with regard to fall detection. For example, wearable devices are always with the user, however they may forget to wear the device. In contrast, non-wearable devices have a limited capture area but the user can safely forget about them. For a full discussion on the advantages and disadvantages of different sensor types refer to recent reviews [19,41].

Fifteen studies used wearable technology and ten used non-wearable, Table 2 shows full details of the devices used in each study. Accelerometers are the most common choice of sensor and have been used in 15 of the studies [17,21,22,23,24,25,29,30,31,32,33,34,36,38,40]. Eight studies tested some form of optical sensor [20,22,26,27,28,30,37,39], making them the most common choice of non-wearable devices. One additional study deployed an optical sensor as part of their system, but this did not record any falls so they could not test it [29].

Studies can be further classified based on whether the device used was capable of processing data on-line and raising an alarm when it detected a fall. Three studies deployed functioning wearable alarm systems [24,33,34], one study deployed a system combining wearable and non-wearable devices [22], no studies deployed an alarm system solely using non-wearable devices. Two of the studies which tested working alarm systems did not store the raw sensor data, only recording when the alarm went off [22,34], one article did not state if the raw sensor data was stored [24]. The raw sensor data can be used for future development and testing, and therefore the favoured approach is to store this data.

The availability of the collected data is important for future work and the direct comparison of approaches. None of the studies used publicly available datasets nor made their real-world fall data publicly available. Two studies [25,40] made use of a subset of the FARSEEING repository, which is available on request. The FARSEEING project is a real-world fall repository project funded by the European Union. Four studies [17,21,23,36] were conducted by members of the FARSEEING project or in collaboration with members, and also used data from the FARSEEING repository. No other studies provide any information on the availability of their datasets.

### 3.3. Number of Participants and Falls, and the Volume of Non-Fall Data

There is a large range in the number of participants included, with most studies using small cohorts. One article did not provide any information on the number of participants [37]. Three studies had just a single participant [31,35,38] and one study [20] used data from only one participant in parts of their analysis. The maximum number of participants was 62 [34] and the median was nine (IQR 4–18).

There was an equally large range in the number of fall events recorded. Two studies included just a single real fall [31,38] and in one of the two datasets used by Aziz et al. [21] only one fall was recorded. The maximum number of falls was 89, which was achieved in two separate studies [23,34]. The median number of falls contained in the datasets used was 17.5 (IQR 8.25–29).

Where reported, the length of the monitoring period varied considerably and comparison is made difficult by the inconsistent choice of reported metrics. Thirteen articles provided the total length of the recorded data, but did not provide details of the proportion where the system was recording participant’s movement (participant in the capture area or wearing the device) [20,21,22,24,26,27,28,30,32,34,35,38,39]. The median length of total recorded data, from studies which provided it, was 592 days (IQR 21–1474). Only three articles provided information on device wear time, in these studies the mean wear times were 8.1 [29], 14.2 [33] and 24 [31] h per day. None of the articles on non-wearable devices provided information on the proportion of time during which participants were in the capture area.

Six articles did not clearly state the time period over which participants were monitored or the amount of data captured, instead they provided the number of extracted non-fall events [17,23,25,36,37,40]. The number of non-fall events used in these studies ranged from 22 [25] to 3466 [23].

### 3.4. Method of Fall Identification and Validation

One of the main challenges in recording real-world falls is ensuring every fall that occurs is identified accurately. How fall events are identified is influenced by both the choice of device and whether the system is capable of raising alarms in real-time. The device used determines the type and detail of information available for retrospective verification of fall times and types. A camera, for example, provides a greater level of information compared to an accelerometer; assuming the video footage is not highly pre-processed, for privacy reasons, before being stored. Where working alarm systems are deployed, all detected falls can be quickly verified, providing additional robustness over a single reporting method such as staff incident reports.

Four studies [22,24,33,34] deployed a functioning wearable alarm system. As the alarm systems were being validated, a second reporting system was still needed to identify falls which did not trigger an alarm. Three of the studies used staff incident reports in addition to the alarm system [22,33,34]. It was unclear what secondary method of fall identification was used in one of the studies [24]. Of the 18 studies which analysed the data retrospectively, three identified falls using staff reports [17,21,39], five used participant self-report [29,30,31,32,38] and ten did not state how falls were identified [20,23,25,26,27,28,35,36,37,40].

Where self-report of falls is used it is important to consider the cognitive ability of participants, especially their memory. Only two of the five studies which used self-report provide results of assessments of cognitive ability [29,30]. Both of these studies used a Mini Mental State Exam [42]. Feldwieser et al. [29] found no signs of cognitive impairment and Gietzelt et al. [30] found that one of their three participants had cognitive impairment, but does not report how they accounted for this.

It is important to consider that reported fall times might not be accurate and that some falls may not be reported, or may be reported by more than one member of staff with different timestamps. This could, for example, be due to delays in completing the report, delays in the faller being discovered, participant recall problems or staff naturally prioritising helping the faller over checking and reporting the time. Only three articles describe methods to check reported fall times [17,21,32]. Two of these [17,21] used datasets from the FARSEEING repository where expert analysis of the sensor signals in combination with fall reports was used to pinpoint the fall signal. Hu et al. [32] reported correlating self-reported fall times with the signals, but provided no details on how this was carried out.

### 3.5. Methods of Data Processing

There are two approaches for testing real-world fall detection systems, the key difference is how the data is prepared. The first approach is based on simply identifying when falls occur in continuous user movement or a stream of sensor data, we call this the continuous data approach. The second approach is based on a fall detector classifying events as either a fall or not a fall, we call this the event based approach. The following sections explain each of these approaches and review their use. In five studies it was unclear which approach was used [20,24,29,30,39].

#### 3.5.1. Continuous Data Approach

The continuous data approach mirrors real-world usage of fall alarm systems where user movement is the input and fall times or alarms are the output. This approach is therefore the primary way of testing deployed fall alarm systems but can also be used for retrospective testing using existing data. The fall detection systems sensors convert movement into a stream of raw data which is then processed by the software component of the system. In this approach all aspects of data processing are part of the fall detection software and are tested as a single unit. To test performance the systems predictions are compared to the actual verified fall times. This comparison allows quantification of the number of true positives (actual and predicted timestamps match), false positives (predicted fall with no actual fall) and false negatives (fall occurred but none was predicted).

True negatives can be quantified if the times when non-falls occurred were recorded, however, non-falls are not defined. In the strictest sense non-falls are everything which is not a fall, but that does not enable their occurrence to be quantified. It is not possible to count when a fall doesn’t occur without arbitrarily dividing the time-series data into events, and counting the events where no fall occurred. Such a method of dividing the data would fall under the event based testing approach. In the continuous data approach any segmenting of the data for processing purposes is part of the fall detection system, not the test procedure.

Six studies used the continuous data approach [22,31,33,34,35,38]. Bloch et al. [22] processed the data using the continuous data approach, and then used an assumption of thirty ‘fall-like’ events per day to calculate a number of true negatives (30 × number of days the sensor was in use). The other five studies did not attempt to quantify TN.

#### 3.5.2. Event Based Approach

The event based approach has its roots in tests using laboratory based simulation datasets. When data is collected in the laboratory a predefined set of movements or events is simulated, the times of these events is known and therefore they can be easily extracted. To test performance all the events must first be labelled as either a fall or not a fall using the record of event times. For each event the label is compared to the software’s predictions allowing a complete confusion matrix to be generated.

In real-world data, events are less clearly defined than in simulated data since there is no complete record of the movements which occurred. The creation of events from real-world data has been based on arbitrary rules rather than identification of the underlying movements of the users. The events are labelled using reported fall times, where no fall occurred the event is considered a non-fall. As this method always yields non-fall events, true negatives can be quantified, unlike in the continuous approach.

Eleven studies used the event based approach [17,21,23,25,26,27,28,32,36,37,40]. The predominant method to create events was based on time windows, where the data is sliced using constant time intervals, for example each 60 seconds of data is one event. However, there is no consensus on what constitutes an event and in practice, a method of reducing the volume of data is often used, for example, to exclude data where no movement was recorded. The time windows can overlap allowing the same data to be processed multiple times, although the rationale for this is not clear.

To create events, one study used 2.5 s windows with a 1.5 s overlap and kept all the events [21]. Two studies divided the data into 60 s windows and used a movement detection algorithm to select events [17,36]. Bourke et al. [23] also used a movement detection algorithm to select events but does not describe the windowing technique. Two studies used the same dataset where the 24 hours prior to each fall was divided into one second windows [27,28]. One study used self-reported wear time to reduce the dataset prior to dividing into windows, but does not provide any details about the windowing technique [32].

Three studies used only a limited section of data from around each fall. Debard et al. [26] divided up the 20 minutes of data prior to a fall into two minute windows. Chen et al. [25] only used data from 20 minutes surrounding each fall and used the section of data up to one second prior to impact as non-fall events. Yu et al. [40] divided the two minutes around each fall into one second windows, removed the one second window where the fall occurred and used the remaining windows as non-fall events.

### 3.6. Definition of Performance Measures and Review of Their Use

#### 3.6.1. Sensitivity

Sensitivity (also known as recall and true positive rate) is the proportion of falls which are correctly detected (Equation (1)). The inverse of sensitivity is miss rate (false negative rate) which quantifies the proportion of falls not detected (Equation (2)). Sensitivity is by far the most commonly reported statistic; it was reported in 18 of the articles [17,20,21,22,23,24,25,26,27,28,32,33,34,36,37,38,39,40] and could be calculated from the information given in the other four [29,30,31,35].

(1) $$\mathrm{Sensitivity}=\frac{TP}{TP+FN}=\frac{TP}{P}$$

(2) $$\mathrm{Miss}\;\mathrm{Rate}=\frac{FN}{FN+TP}=\frac{FN}{P}=1-\mathrm{Sensitivity}$$

#### 3.6.2. Specificity

Specificity (also known as true negative rate) is the proportion of non-fall events which are correctly detected (Equation (3)). It quantifies the ability to avoid false positives (false alarms). The inverse of specificity is false positive rate, which is the proportion of non-fall events mistakenly detected as falls (Equation (4)). Nine articles reported specificity [17,21,22,23,24,26,32,36,40] and two reported false positive rate [36,37]. It is unclear whether Chen et al. [25] reported specificity or false positive rate, as the reported number of TN and FP suggest that what they report as specificity is in fact false positive rate. Specificity could be calculated from the information provided in a further two of the studies [27,28].

(3) $$\mathrm{Specificity}=\frac{TN}{TN+FP}=\frac{TN}{N}$$

(4) $$\mathrm{False}\;\mathrm{Positive}\;\mathrm{Rate}=\frac{FP}{FP+TN}=\frac{FP}{N}=1-\mathrm{Specificity}$$

#### 3.6.3. False Positive Rate over Time

False Positive Rate over Time (FPRT) has become a popular measure in real-world tests of fall detection. This measure provides information on the frequency of false alarms. Twelve articles report the number of false positives either per hour or per day [17,20,21,28,29,30,31,33,35,36,38,39] and it could be calculated from the information provided in seven others [24,25,26,27,32,34,37].

#### 3.6.4. Precision

Precision (also known as positive predictive value) is the proportion of alarms which are true falls (Equation (5)). It therefore provides the probability that an alarm will be an actual fall and not a false alarm. For example, a precision of 0.5 means that half of alarms will be actual falls, and half will be false alarms (1 false positive for every detected fall). Eight articles reported precision [17,22,24,26,27,28,34,40] and it could be calculated from the information provided in all of the other articles.

(5) $$\mathrm{Precision}=\frac{TP}{TP+FP}$$

#### 3.6.5. Negative Predictive Value

Negative Predictive Value (NPV) is the proportion of events classified as non-falls which are true non-fall events (Equation (6)). NPV therefore provides information about the ability to correctly classify non-fall events. NPV will be high if a system correctly ignores many times more non-fall events than the number of falls it fails to detect. Therefore, for false negatives to have any notable effect, the number of falls and non-falls must be approximately equal. However, in real-world fall data falls are usually much less frequent than non-fall events, which limits the insights yielded from NPV as systems typically score over 0.99 out of 1 [17,22,24]. Three articles reported NPV in their results [17,22,24]. NPV could also be calculated from the information provided in eleven of the other articles [21,23,25,26,27,28,32,34,36,37,40].

(6) $$\mathrm{Negative}\;\mathrm{Predictive}\;\mathrm{Value}=\frac{TN}{TN+FN}$$

#### 3.6.6. Accuracy

Accuracy is the proportion of predictions which were correct (Equation (7)). Accuracy is a measure which summarises the whole confusion matrix in a single value. Accuracy’s major limitation is the inability to handle imbalanced datasets, for example, in real-world fall data where there are many more non-fall events than falls. Similar to NPV, accuracy is dominated by the larger group and the effect is proportional to the size of the imbalance. Therefore, in real-world fall detection studies, accuracy is skewed towards the correct detection of non-fall events over the correct detection of falls. For example, in eight of the algorithms tested by Bagala et al. [17] the accuracies were greater than 0.9 with sensitivities below 0.6, in one case an accuracy of 0.96 with a sensitivity of 0.14. Four articles reported accuracy [17,23,25,37] and it could be calculated from the results provided in seven of the other articles [21,24,26,27,28,36,40].

(7) $$\mathrm{Accuracy}=\frac{TP+TN}{P+N}$$

#### 3.6.7. F-Measure

F-measure (also known as F-score) is the harmonic mean of sensitivity and precision (Equation (8)). F-measure, therefore, considers all outcomes except true negatives (non-falls). In fall detection, the priorities are detected falls (TP), missed falls (FN) and false alarms (FP). F-measure considers all of these outcomes and therefore provides a good overview of performance. No articles report a value for F-measure, however it could be easily calculated from their results as eight articles [17,22,24,26,27,28,34,40] reported both sensitivity and precision and all but two [32,39] reported enough information to calculate both sensitivity and precision.

(8) $$F-\mathrm{measure}=2\times \frac{\mathrm{Precision}\times \mathrm{Sensitivity}}{\mathrm{Precision}+\mathrm{Sensitivity}}$$

#### 3.6.8. Informedness

Informedness (also known as Youden’s J Statistics or Youden’s Index) is a statistic which combines sensitivity and specificity (Equation (9)). It is the probability that predictions are informed versus a pure guess. Informedness is linked to the proportion of cases classified correctly. However, unlike accuracy, it is robust to an imbalance in the number of fall and non-fall events. This is achieved through equal weighting of sensitivity and specificity which are in turn the proportions of falls detected and non-falls correctly ignored. The value ranges from negative one to positive one. Zero indicates predictions are no better than guessing, positive one indicates perfect predictions and negative one indicates all predictions are the opposite of the true value. In cases where the value is negative, the output classes can simply be swapped over. One study reported informedness [36], however, 12 other articles reported both sensitivity and specificity or false positive rate, or the information necessary to calculate them [17,21,22,23,24,25,26,27,28,37,40], so informedness could be calculated from their results.

(9) Informedness=Sensitivity+Specificity−1

#### 3.6.9. Markedness

Markedness is a statistic which combines precision and NPV (Equation (10)). Markedness is linked with the proportion of predictions which are correct. It combines the proportion of correct positive and negative predictions with equal weighting and is therefore unaffected by imbalance in the number of positive and negative predictions. As with informedness, the result is a value between negative and positive one. No articles reported markedness, but twelve did report enough information for markedness to be calculated [17,21,22,23,24,25,26,27,28,36,37,40].

(10) Markedness=Precision+NPV−1

#### 3.6.10. Matthews Correlation Coefficient

Matthews Correlation Coefficient (MCC) is the geometric mean of informedness and markedness (Equations (11) and (12)). It should be noted that Equation (11) only works if informedness and markedness are both positive, Equation (12) works in all cases. MCC considers both the proportion of events classified correctly and the proportion of correct predictions and is therefore robust to imbalanced datasets. The result is a value between negative and positive one as with both informedness and markedness. None of the articles reported MCC, enough information to calculate MCC was given in 14 articles [17,21,22,23,24,25,26,27,28,32,34,36,37,40].

(11) $$\mathrm{MCC}=\sqrt{\mathrm{Informedness}\times \mathrm{Markedness}}$$

(12) $$\mathrm{MCC}=\frac{TP\times TN-FP\times FN}{\sqrt{\left(TP+FP)(TP+FN)(TN+FP)(TN+FN\right)}}$$

#### 3.6.11. Receiver Operating Characteristic Curve

A Receiver Operating Characteristic (ROC) Curve is a plot of sensitivity versus false positive rate as the primary threshold of the classifier is adjusted. ROC curves can therefore be used to understand the trade-off between sensitivity and false positive rate and optimise a primary threshold. There could be debate as to which balance of sensitivity and false positives is optimal, therefore a ROC curve provides useful insight. However, it is difficult to compare systems robustly based on a curve. Consequently, it is in the optimisation where ROC curves are best used, rather than final results, as only the optimised version will be deployed.

ROC curves can be reduced to a single number by calculating the area under the curve (AUC). AUC has been found to be a poor measure for comparing classifiers, particularly where the sample size is small [43,44,45]. Two studies have used ROC analysis and reported AUC [23,36].

#### 3.6.12. Precision-Recall Curve

A precision-recall (PR) curve is similar to a ROC curve, the difference is that precision is used instead of false positive rate and the term recall is used in place of sensitivity. PR curves are preferred over ROC curves when there is a large imbalance in the data [46]. Calculating AUC for PR curves is more challenging than for ROC curves as precision does not increase linearly, meaning linear interpolation yields incorrect results [46]. Two studies reported PR AUC [27,28], although it is unclear how PR AUC was calculated in these studies.

## 4. Discussion

This is the first review to be conducted on the methods used to evaluate real-world performance of fall detection systems. Ensuring a sound method is critical for meaningful results, therefore reflecting on the way studies are conducted and seeking improvements to the method is vital in emerging areas of research where no consensus has yet been reached. The real-world testing of fall detection systems is currently in its infancy and this is reflected in our findings. The method is highly variable across studies, which makes comparing the results difficult if not impossible. The following three sections discuss the key issues and make recommendations for future studies.

### 4.1. Data Collection and Preparation

One major aspect which leads to variation between studies is the participant groups and the differences in the movements and behaviours captured by the sensor systems. If insufficient detail is gathered about participants it is challenging to reproduce the findings as differing results could be due to differing participant characteristics. In addition, one may want to collect new data comparable to that used in a previous study for the purpose of comparing the performance of a new system using different sensors with previously tested systems. Information gathered about participants was both inconsistent and insufficient to allow the data collection to be reproduced.

A comprehensive consensus process has previously been carried out by the FARSEEING consortium [47]. As part of the consensus process the group identified a minimum set of clinical measures which they deemed essential for the interpretation of real-world fall data. The measures included age, height, weight, gender, fall history, assistive device use as well as assessments of mobility, cognitive impairments and visual impairments. None of the reported studies have implemented these recommendations.

Cognitive and mobility tests provide useful information about fall risk and the likelihood of false positives caused by events such as ‘falling into a chair’ or improper use of the device. Compared to standard metrics such as age, height and weight, assessments of mobility and cognition provide a much deeper insight into participant’s fall risk and movement characteristics. Therefore, standardised cognitive and mobility assessments should be prioritised. Deeper insights into participant’s movements could be achieved though continuous profiling using activity monitoring software to process the recorded dataset. However, development and validation of activity monitoring software may be a barrier unless an existing activity monitoring system is used for the data collection. Where such profiling is possible details should be reported to enhance the interpretation of results.

Another critical aspect of the test is the size of the dataset. Currently, the datasets used are generally small, have been collected with a low number of participants and contain only a few falls. Small datasets reduce the validity of the test and hinder reproducibility. Where the dataset is small either due to few participants, a low incidence of falls or both, it is possible that only a limited subset of movements and fall types were captured. In such cases comparisons of results to tests of other systems is difficult as the dataset may be the main cause of differences in reported performance. Further, the generalisability of results is questionable where the sample size is small. The small datasets are one factor which makes it difficult to understand which systems perform the best and therefore where future development should focus. The other main factors are the different populations recruited for studies and the limited insights into how this effects the fundamental aspect of the data—the movements captured.

Due to the known challenges in recording fall signals, the only feasible way for most researchers to gain access to a large number of fall signals is through collaboration. In addition, if systems are tested using the same data, the results are directly comparable. Therefore, large shared test datasets are needed to allow the performance of fall detection software to be compared. To facilitate the sharing of datasets, the FARSEEING consortium have established a data repository which currently contains over 300 fall signals [48]. However, more studies are needed to generate datasets that can be added to the repository and used for robust testing of devices and development of improved software.

Even with shared data, there is still an issue of how to ensure all fall signals are accurately identified. We have identified that the method used to identify the fall signals is poorly described in published studies, leaving a large gap in our understanding of how the dataset was prepared. The current prevailing method to identify fall signals is expert signal analysis to verify participant or staff reported fall times. There is a risk that not all falls are reported, leading to real falls being included as non-fall data. Expert signal analysis cannot overcome the issue of under reporting, but does at least give greater confidence that inaccurate reported times were corrected and all included fall signals were real falls.

Expert signal analysis, while clearly better than no verification, could lead to bias. Currently there is an insufficient understanding of fall signals due to a limited number of recorded falls and a lack of research into the profile of the signals. Our limited understanding could lead to atypical falls not being verified and thus excluded. There is a risk that systems are designed to detect certain signal profiles as falls and only these profiles are being verified as falls. Therefore the results could be artificially improved through restricting the test data.

Unless a gold standard fall reporting system is used, such as video analysis, studies will be limited in their ability to verify fall signals, under reporting of falls will remain a concern and there is a risk of bias in the verification process needed to compensate for the inaccuracies of the ‘silver standard’ reporting system. The current lack of standardised method or gold standard, and the lack of reporting how fall signals were identified and verified, inhibits understanding of results. A consensus is needed on the process for fall signal identification and studies should clearly report their methods.

### 4.2. Data Processing

Two approaches were identified for preparing sensor signals for fall detection system testing and we named these the continuous data approach and the event based approach. Both approaches have issues surrounding what constitutes a non-fall. In the continuous data approach the issue is centred around the definition and identification of non-falls. In the event based approach non-fall events can be defined as any event which is not a fall. However, events could be defined as anything which is either a fall event or non-fall event, and since falls are defined, the issue returns to what constitutes a non-fall.

The strictest definition of non-falls as everything which is not a fall is not particularly useful. This definition does not allow non-falls to be quantified in the continuous data approach and provides no indication of how the data should be divided into events for the event based approach. A more helpful concept is that of fall-like movements, a subset of non-falls which share characteristics with falls. The FARSEEING consortium defined a fall as “an unexpected event in which the person comes to rest on the ground, floor or lower level” [49]. A fall-like movement could therefore, by removing the unexpected clause, be defined as “any event in which the person comes to rest on the ground, floor or lower level”.

With a definition for fall-like events these could be recorded, at least theoretically, in the same manner as falls and therefore, allow true negatives to be quantified robustly. In reality it is not feasible for a researcher to record the times of all fall-like movements in the same way that falls are recorded, due to the vast quantity which would occur. An automated system would be more practical, although it is unlikely to be easier to develop automated fall-like detection than automated fall detection systems. Consequently, researchers must consider if the development of fall-like movement detection systems is worth the investment, simply to extend the testing of fall-detection systems. Given that a robust evaluation of fall detection systems can be achieved without the need for true negatives, and hence non-fall or fall-like movements, we suggest that automated fall-like movement detection is unlikely to bring benefits which outweigh the required investment.

### 4.3. Performance Measures

It is challenging to compare results across studies or determine the current state-of-the-art due to disparity in the choice of measures reported and challenges calculating unreported measures. The measures used to report and interpret performance vary widely across studies and not all studies report the basic results from which all measures can be calculated (TP, FP, FN and TN). Where TP, FP, FN and TN are not reported these can only be estimated, due to rounding of the reported results. Using one of the tests reported by Bourke et al. [23] as an example, the number of FP could be any value between 18 and 51 based on the reported specificity of 0.99 with 3466 total non-falls. To facilitate the calculation of additional measures, future studies should report TP, FP, FN and TN if these can be calculated robustly and are used in the calculation of the reported performance measures.

In addition to reporting enough information to allow further measures to be calculated, it is important that the headline measures give a true reflection of performance and allow robust comparisons to be made with other systems. Sensitivity has been a mainstay in previous studies, it is an important aspect of system performance. Sensitivity only quantifies the ability to detect falls, it does not consider false positives. The question is therefore which measure to pair sensitivity with to provide understanding of the ability to avoid false positives. In addition, a single combined measure which considers both aspects is important in order to understand the overall level of performance.

Specificity has been the most common choice of measure to quantify the ability to avoid false alarms in laboratory based testing [19] and it has remained a common choice in real-world tests. Specificity considers how well non-fall events are classified, it could therefore be considered sensitivity’s natural counterpart. The weakness of specificity in the context of real-world fall detection is the reliance on non-falls, which are poorly defined and troublesome to identify.

The need for researchers to design or select methods for non-fall identification opens up a considerable possibility of bias. A method could be used which suits the specific system and dataset causing distortion of the results and hindering comparisons with other systems. In the case of specificity, the difficulty of the test is very much determined by the definition of a non-fall; the more inclusive the definition, the more non-fall events and therefore the higher the score for the same number of false positives. This effect can be seen in the study of Bourke et al. [23], where tests were conducted twice using different definitions of non-falls. With the most restrictive definition of non-falls, specificity ranged from 0.83 to 0.91. With the more open definition, specificity was consistently 0.98 or greater. Expanding the definition includes more movements which are less fall-like, thus it creates an easier test.

It is hard to prevent bias in selecting a definition of non-falls as it is likely unintentional. One solution is to remove the need to select a method on a study by study basis, however, standardising the method is challenging. Since there is currently no clear way to standardise non-fall identification, the best option may simply be to avoid them altogether. A solution might be standard publicly available datasets, with an agreed method to identify non-fall events. In such a case, the results are comparable to each other, but not to other studies using other datasets or methods.

Using standard data is challenging due to the vast array of sensors which could be used and the huge number of combinations. It is simply not possible to have a single dataset used to test all systems. Furthermore, it seems impossible to identify all types of relevant non-fall movements needed for a universal standard dataset. Any measures which rely on non-falls (specificity, NPV, accuracy, informedness, markedness, MCC and ROC AUC) are subject to the above problems and therefore should not be used as a primary measure. Where measures reliant on non-falls are used the methods should be described in detail and their limitations should be made clear to avoid confusion and misinterpretation.

The issues surrounding non-falls substantially reduces the options for quantifying the ability to avoid false positives and gauge overall performance. There are four possible measures which do not rely on non-falls, these are FPRT, precision, F-measure and PR AUC.

FPRT is a useful measure to understand the frequency of false alarms, however differences in the datasets affect the calculation. Wear time or time in the capture area must be considered, as false positives will, most likely, be far lower when the device is not in use. Another consideration is which hours of the day the device is in use; false positive rate during night time hours would be very different to day time hours. Reporting of times when the device was monitoring participants was found to be inadequate. Of the 11 articles which reported FPRT only two clearly reported wear time or time in the capture area [29,33] and none reported any details on the distribution of this time throughout the day.

Our findings suggest that there is a lack of an agreed and clearly defined method to calculate FPRT. Only one study clearly states that FPRT was calculated using solely the time a participant was being monitored by the device [33]. None of the other studies appear to have taken usage time into account when calculating FPRT. If usage time is not considered or reported it is unclear what extent device usage, or lack thereof affected the result. An unused system is unlikely to produce false positives. The issues in identifying wear time or time in the capture area could make FPRT an unreliable measure to compare across studies. Although users and clinicians may find the rate of false positives over time useful, it might be better to use a rate of something other than time.

Precision is an alternative to specificity and FPRT, it quantifies the false positives (FP) in relation to detected falls (TP). TP and FP should, for any reasonable level of performance, be in the same order of magnitude, therefore precision is resilient to the imbalance in the data. Further, the ratio between TP and FP is unlikely to be notably affected by usage time, if a device is used half of the time, TP and FP would be expected to be half compared to full device usage. Therefore, compared to FPRT, precision is far less affected by device usage, or lack thereof. The proportion of fall predictions which were true falls could be more useful than FPRT since frequent false positives may be acceptable to a frequent faller, assuming the falls are detected. Precision should be the primary measure of the ability to avoid false positives.

Sensitivity and precision together quantify the ability to detect falls and avoid false alarms, therefore providing a complete portrayal of performance. In addition to sensitivity and precision it is important to have a single measure which can quantify the trade-off between them. PR AUC is one possible option, however it considers the performance of multiple sub-optimum versions of the system as the system’s parameters are adjusted. Since only the optimised system can be deployed, it is the optimised version which should be the focal point of the evaluation. F-measure, the harmonic mean of sensitivity and precision, appears to be the most suitable single measure for objective comparison. This trio of measures has two major advantages in robustness: (1) it does not rely on non-falls and (2) it is resistant to issues surrounding wear time and time in the capture area. Future studies should report sensitivity, precision and F-measure, and F-measure should be used as the standard for comparing systems.

## 5. Summary and Conclusions

As focus in fall detection performance evaluation shifts from simulated to real-world fall data, one must consider if the approach used for evaluating on simulations is optimum for real-world data. Through examining the published articles on evaluation of real-world fall detection, two issues have become apparent:

1. The approaches to quantifying performance are inconsistent and many studies use measures which provide limited representation of performance.
2. The number of falls is generally small and study populations are diverse, making comparison between the datasets and results difficult.

It is critical that a consensus is reached on the most appropriate method to evaluate real-world performance of fall detection systems.

To address the issues with the datasets there needs to be greater collaboration and sharing of data. The FARSEEING consortium have made substantial steps to facilitate data sharing and have recorded over 300 falls through collaboration between six institutions [48]. Six of the 22 studies published to date have used parts of this data to develop or test approaches to fall detection [17,21,23,25,36,40], highlighting the importance of this data. However, further work is still needed to grow the volume of available data, record more falls, improve standardisation and further develop fall detection technology. Only through collaboration will the collection of a dataset large enough for robust development and testing become possible.

To address the issues surrounding how performance is quantified studies should avoid the need for non-falls. The concept is poorly defined and standardisation seems to be extremely problematic. The concept of non-falls is only needed to allow the calculation of measures such as specificity and accuracy, both of which are common in simulation based studies [19]. However, quantification of the difference in false alarm rate between simulated and real-world tests is not possible due to the disparity of the data. Therefore, traditional measures such as specificity and accuracy are of little value. Continued use of these traditional measures may lead to confusion and improper interpretation of performance. Measures which do not depend on non-falls should be used instead of these traditional measures. Sensitivity and precision should be the cornerstones of the evaluation with F-measure used for the objective comparison of systems.

## Figures and Tables

**Table 1:** Example Search Strategy for PubMed.

	fall*-detect*[Title/Abstract] OR fall*-sensor*[Title/Abstract] OR fall*-alarm*[Title/abstract]
AND	real-world[Title/Abstract] OR real-life[Title/Abstract] OR free-living[Title/Abstract] OR community-dwelling[Title/Abstract] OR home-dwelling[Title/Abstract] OR domestic-environment[Title/Abstract] OR long-term-care[Title/Abstract] OR care-home[Title/Abstract] OR nursing-home[Title/Abstract] OR hospital[Title/Abstract]

**Table 2:** Summary of papers evaluating fall detection systems using real-world falls.

Author	Participant Group	Additional Information	Device Type	Number of Participants	Number of Falls	Quantity of Non-Fall Data and Method of Preparation		Performance Measures
Aziz [21]	Residents of a long-term care facility who had experienced at least one fall in the previous year	Age, mobility assessment	Accelerometer	9	1	214 h	Data were divided into 2.5 s time windows with a 1.5 s overlap. The 30 s of data following a fall event were ignored.	**Sensitivity**, Specificity, **FPRT**, TP, FP, FN
	Patients at a hospital geriatrics department with Progressive Supranuclear Palsy	Age	Accelerometer	10	9	178 h		
Bagala [17]	Patients with Progressive Supranuclear Palsy	Age, gender, height, weight	Accelerometer	9	29 the number from each group was not provided	A total of 168 h from seven of the participants. Recordings were divided into 60 s windows and only the 1170 windows where max(RSS)−min(RSS)>1.01 g were included		**Sensitivity**, **Specificity**, **FPRT**, Precision NPV, Accuracy
	Community dwelling older adult	None	Accelerometer	1				
Bloch [22]	Patients at a geriatric rehabilitation ward with an identified risk of falling	Age	Working alarm composed of an accelerometer and infrared sensor	10	8	A total of 196 days. Data was processed on-line and the analysis compared the alarm times to reported fall times. Assumed 30 fall like events per day to estimate of the number of non-fall events.		**Sensitivity**, **Specificity**, Precision, NPV, TP
Bourke [23]	Patients at a geriatric rehabilitation unit	None	Accelerometer and gyroscope	42	89	A total of 3466 events extracted using a dynamic detection algorithm and further reduced to 367 events where: max(RSS)>1.05 g Total length of recorded data was not given.		**Sensitivity**, **Specificity**, Accuracy, ROC AUC
Chaudhuri [24]	Community dwelling older adults	None	Working alarm consisting of an accelerometer, magnetometer, and gyroscope	18	14	A total of 1452.6 days. Details of data preparation not given.		Sensitivity, Specificity, Precision, NPV, Confusion Matrix
Chen [25]	Community dwelling older adults living in geriatric rehabilitation centres	Age, gender, height, weight	Accelerometer	22	22	A total of 22 events. Only data from a 1200 s window around the falls was used, data up to 1 s before each fall were used as non-fall events.		Sensitivity, FPR, Accuracy, Confusion matrix
Debard [26]	Older adults	Age	Camera	4	25	A total of 14,000 h. Only data for the 20 min up to and including the falls were used, this was divided into 2 min windows.		**Sensitivity**, Specificity, **Precision**, Confusion matrix
Debard [27]	Older persons (two community dwelling, one in a nursing home and four in assisted living), two of which did not fall and were excluded	Age, mobility assessment, walking aid use	Camera	7	29	Over 21,000 h recorded. Only data from the 24 h prior to each fall were used which was divided into 1 s windows.		Sensitivity, Precision, PR Curve, PR AUC, TP, FP, FN
Debard [28]	Older persons (two community dwelling, one in a nursing home and four in assisted living), two of which did not fall and were excluded	Age, mobility assessment, walking aid use	Camera	7	29	Over 21,000 h recorded. Only data from the 24 h prior to each fall were used which was divided into 1 s windows.		**Sensitivity**, Precision, PR Curve, **PR AUC**, TP, FP, FN, **FPRT**
Feldwieser [29]	Community dwelling older adults	Age, height, weight, mobility assessments, cognitive assessments	Accelerometer	28	12	A total of 1225.7 days (average daily user wear time 8.1 ± 4.8 h). Details of data preparation not given.		**TP**, FP, FPRT
Gietzelt [30]	Older adults with recurrent falls	Age, gender, mobility assessments, cognitive assessments	Accelerometer and camera	3	4	A total of 10 days. Details of data preparation not given.		TP, FPRT
Godfrey [31]	Older adult with Parkinson’s disease	Age, BMI, balance assessment	Accelerometer	1	1	A total of 7 days. No preparatory steps.		TP, FPRT
Hu [32]	Community dwelling older adults with a history of falls	Age, gender, height, weight	Accelerometer and Gyroscope	5	20	A total of 70 days, divided into sliding windows. Window size was varied from 5 to 30 min.		Sensitivity, Specificity
Kangas [33]	Residents of elderly care units	Age, gender, mobility assessments, cognitive assessments	Accelerometer	16	15	A total of 1105 days (average daily user wear time 14.2 ± 6.3 h). Data processed on line, 14 s raw acceleration data where recorded when acceleration of all three axes fell below 0.75 g.		**Sensitivity**, **FPRT**, TP, FP
Lipsitz [34]	Residents of a long-term care facility who had at least once in the previous 12 months	Age, gender, height, weight, BMI, prevalence of 21 comorbidities	Working alarm system using an accelerometer	62	89	A total of 9300 days. Working alarm, raw sensor data not stored, analysis compared the alarm times to reported fall times.		Sensitivity, Precision, TP, FP, FN
Liu [35]	Older adult	None	Doppler radar	1	6	A total of 7 days. No preparatory steps.		TP, FPRT
Palmerini [36]	Patients with Progressive Supranuclear Palsy staying in a geriatric rehabilitation unit	Age, gender	Accelerometer	1	12	A total of 168 h from four of the participants. Recordings were divided into 60 s windows and only the 1170 windows where max(RSS)−min(RSS)>1.01 g were included		Sensitivity, Specificity, FPR, FPRT, Informedness, ROC Curve, **ROC AUC**, FP
	Community dwelling patients with Progressive Supranuclear Palsy	Age, gender	Accelerometer	6	16			
	Community dwelling older adult	Age, gender	Accelerometer	1	1			
Rezaee [37]	Nursing home residents	None	Camera	Not given	48	A total of 163 normal movements extracted from video sequences totalling 57,425 frames. Details of identification not given.		**Sensitivity**, **Accuracy**, **FPR**, Confusion matrix
Skubic [20]	Residents of an older adult independent living facility	Age, gender	Doppler radar	1	13	10 days	Details of data preparation not given for any of the datasets.	Sensitivity, FPRT, TP, FP
	Residents of an older adult independent living facility	Age, gender	Kinect	16	9	3,339 days		
	Resident of an older adult independent living facility	Age, gender, mobility device use	Kinect	1	142	601 days		
	Residents of assisted living apartments	Gender	Kinect	67	67	10,707 days		
Soaz [38]	Older adult	Age, gender	Accelerometer	1	1	3.5 h	No preparatory steps.	Sensitivity, **FPRT**, FP
	Older adults	Age, gender	Accelerometer	14	0	996 h		
Stone [39]	Residents of an older adult independent living facility	Age, gender	Kinect	16	9	A total of 3339 days. Device only stored data for periods where motion was detected.		Sensitivity, FPRT
Yu [40]	FARSEEING data used previously in [17,23] no further details provided	None	Accelerometer	22	22	A total of 2618 normal activities extracted as 1 s windows from the 2 min surrounding the fall signals.		**Sensitivity**, **Precision**, Specificity

Notes: Performance measures reported in the articles abstract are shown in bold. Where a working alarm system was tested this is stated in the Device Type column, otherwise the test was carried out off-line, using the collected dataset. Soaz [38] focused on estimating the false alarm rate, however one real fall was recorded by chance and was included. RSS = Root Sum of Squares; FPRT = False Positive Rate Over Time; NPV = Negative Predictive Value; ROC Curve = Receiver Operating Characteristic Curve; ROC AUC = Area Under ROC Curve; PR Curve = Precision Recall Curve; PR AUC = Area Under Precision Recall Curve; TP = True Positives; FP = False Positives; FN = False Negatives; TN = True Negatives.

## Abbreviations

The following abbreviations are used in this manuscript:

P	Positive cases
N	Negative cases
TP	True Positives
FP	False Positives
FN	False Negatives
TP	True Positives
NPV	Negative Predictive Value
FPRT	False Positive Rate over Time
MCC	Mathews Correlation Coefficient
ROC	Receiver Operating Characteristic
PR	Precision-Recall
AUC	Area Under Curve
RSS	Root Sum of Squares
IQR	InterQuartile Range
